# Accessory Breast Apocrine Carcinoma: Report of a Rare Case

**DOI:** 10.7759/cureus.104821

**Published:** 2026-03-07

**Authors:** Yoshihiko Mishima, Tatsuhiro Nakagawa, Hikaru Imai, Fumi Sakurai, Toshihiro Saito

**Affiliations:** 1 Plastic Surgery, Osaka Rosai Hospital, Osaka, JPN

**Keywords:** accessory breast carcinoma, apocrine carcinoma, axillary lymph node dissection, lymph node metastasis, triple-negative breast cancer (tnbc)

## Abstract

Axillary apocrine carcinoma may arise from cutaneous adnexal glands or breast tissue, including accessory breast tissue. Accessory breast carcinoma is rare, and apocrine carcinoma represents an uncommon histologic subtype of breast cancer. For accessory breast carcinoma, wide local excision and lymph node dissection is generally recommended. Postoperative therapy, including endocrine therapy, is considered following treatment strategies for breast cancer. We report an extremely rare case of accessory breast apocrine carcinoma (ABAC). To the best of our knowledge, this is the first case documented in the English-language literature. A 57-year-old woman presented with an enlarged cutaneous tumor in the right axilla. Initial excision revealed apocrine carcinoma arising from the cutaneous adnexal glands or accessory breast tissue. Imaging demonstrated axillary lymph node metastasis, and the patient subsequently underwent wide local excision with axillary lymph node dissection. Histopathological examination confirmed accessory breast tissue adjacent to the carcinoma, leading to a diagnosis of ABAC. The tumor showed a triple-negative phenotype, and postoperative radiation therapy was administered. At the five-month follow-up, no recurrence or lymphedema was observed.

## Introduction

Axillary apocrine carcinoma may arise from cutaneous adnexal glands or breast tissue, including accessory breast tissue [1,2]. Accessory breast tissue is a congenital anomaly most commonly found in the axilla [3]. Similar to normal breast tissue, it has the potential to develop carcinoma, although this remains rare [4].

Apocrine carcinoma is an uncommon histologic subtype of breast cancer, accounting for approximately 1% of cases [5-7]. Because of their rarity, subtype-specific treatment strategies have not been established; therefore, breast apocrine carcinoma is generally treated in the same manner as invasive ductal carcinoma (IDC), and its prognosis is known to be more favorable than that of IDC [6,7].

Although no standardized treatment protocol exists for accessory breast carcinoma, wide local excision with lymph node dissection is generally recommended [4,8]. Postoperative therapy, including endocrine therapy such as tamoxifen or aromatase inhibitors, is considered following treatment strategies for breast cancer [4,8]. As endocrine therapy is not typically administered for adnexal carcinoma, misdiagnosis of the tumor origin may lead to inappropriate treatment strategies.

Here, we report an exceptionally rare case of accessory breast apocrine carcinoma (ABAC). To the best of our knowledge, this is the first documented case of ABAC in the English-language literature.

This case was previously presented as a meeting abstract at the 139th Meeting of the Kansai Society of Plastic and Reconstructive Surgery on November 15, 2025.

## Case presentation

**Figure 1:**
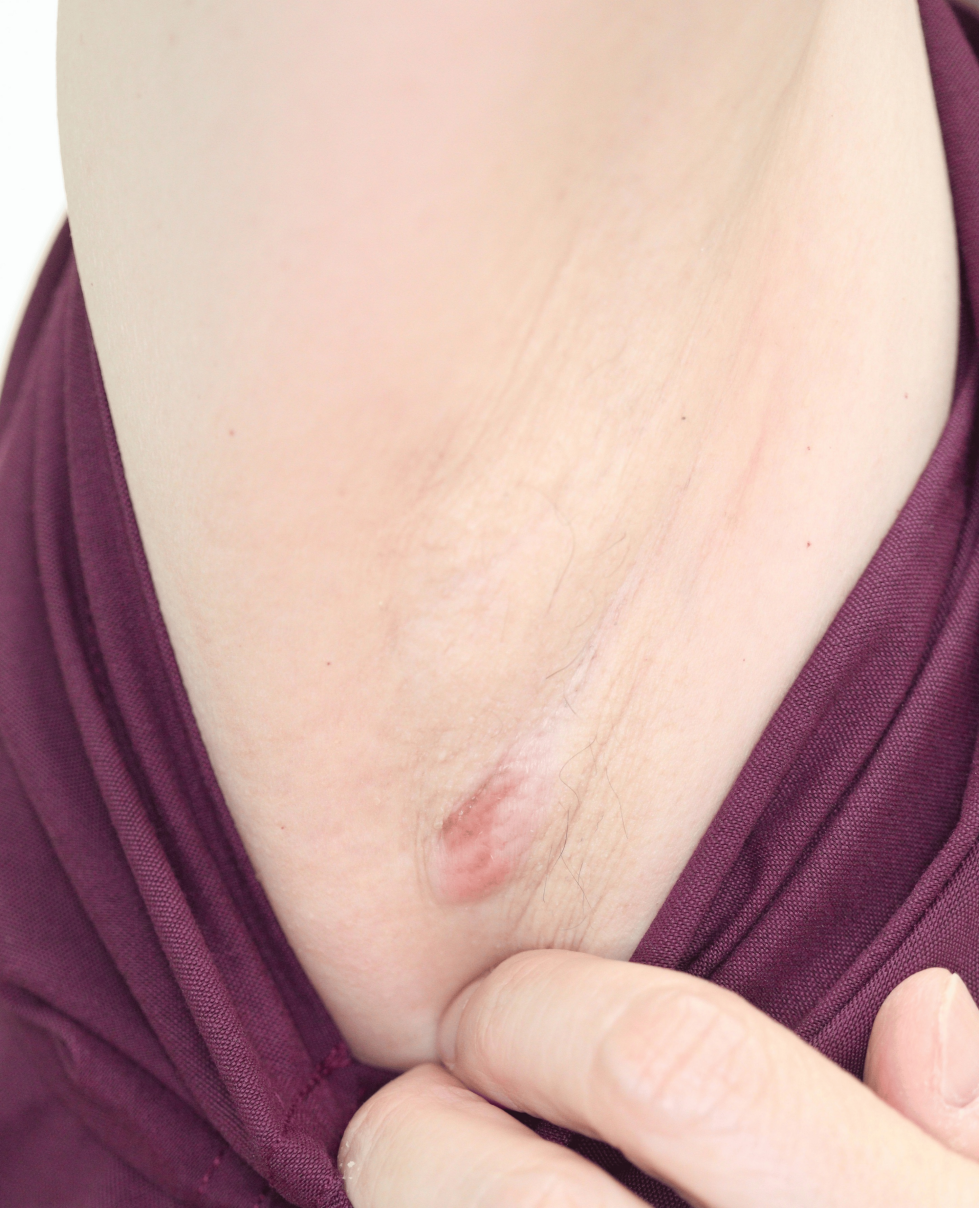
Initial examination A firm, non-tender, reddish cutaneous tumor measuring 1 × 2 cm was observed in the right axilla.

A 57-year-old woman presented with an enlarged cutaneous tumor in the right axilla. She had noted a small mass in the same location for more than 10 years, which had progressively increased in size and developed erythema over the past six months. On examination, the axillary tumor was firm, non-tender, and measured 1 × 2 cm (Figure 1). Additionally, a soft and freely movable subcutaneous mass was observed on her right shoulder. Laboratory findings revealed no clinically significant abnormalities. Her medical and family histories were unremarkable. Magnetic resonance imaging revealed no distinct lesion in the right axilla but demonstrated a 5-cm benign lipoma on the right shoulder.

**Figure 2:**
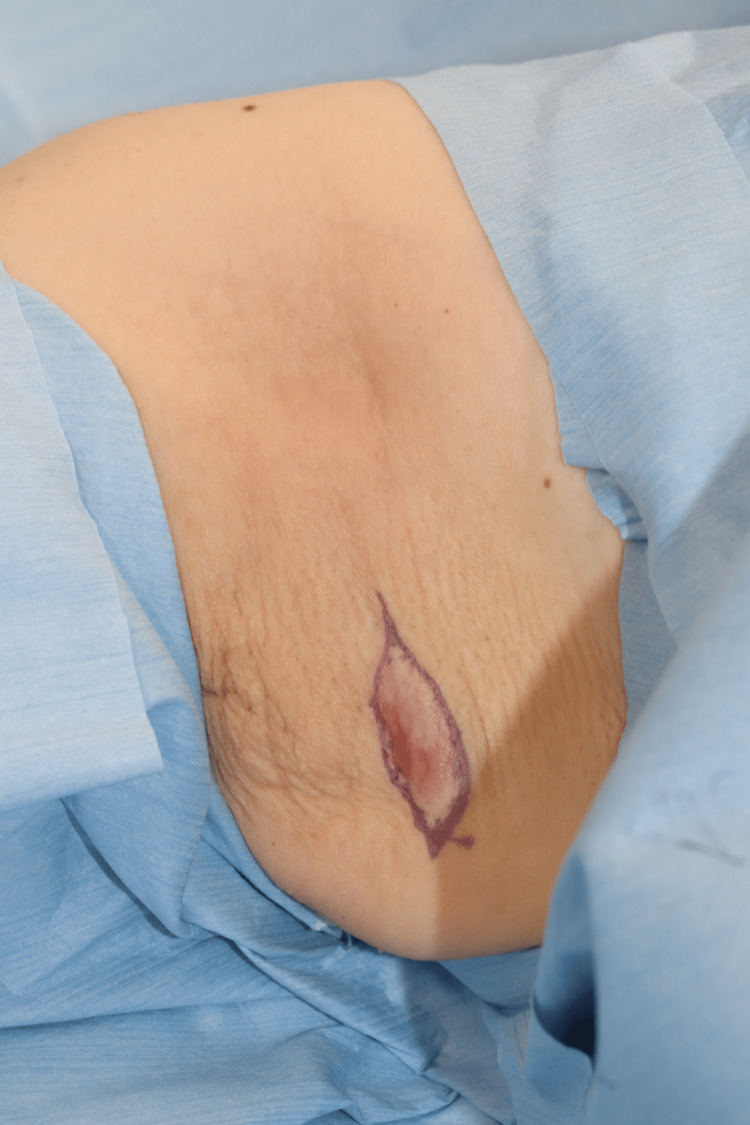
First surgery The axillary tumor was excised in a spindle-shaped fashion under local anesthesia.

**Figure 3:**
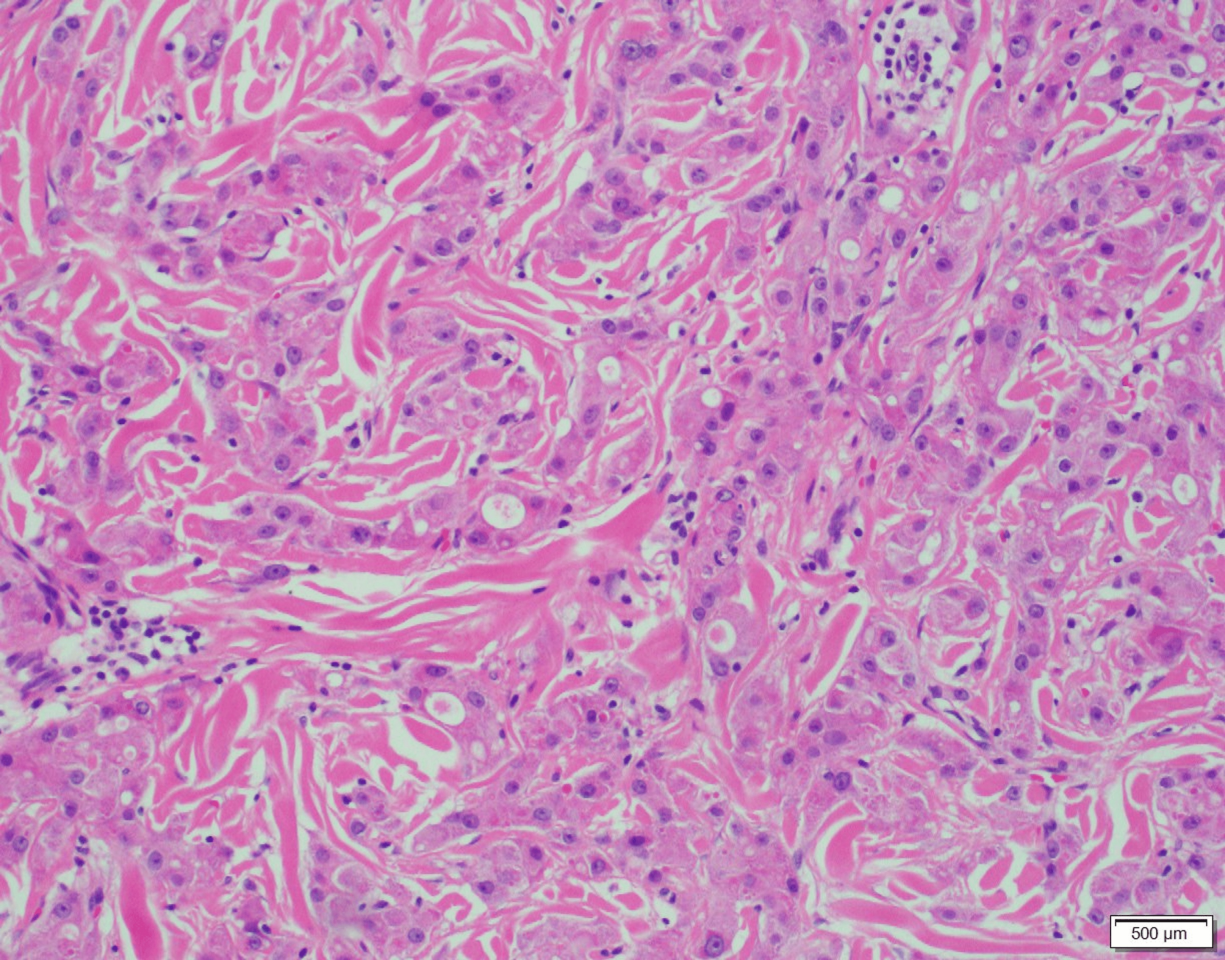
Hematoxylin & eosin ×100 Polygonal atypical cells with eosinophilic granular cytoplasm and large nuclei containing prominent nucleoli proliferated invasively in trabecular and small nest-like patterns, occasionally forming tubular structures.

**Figure 4:**
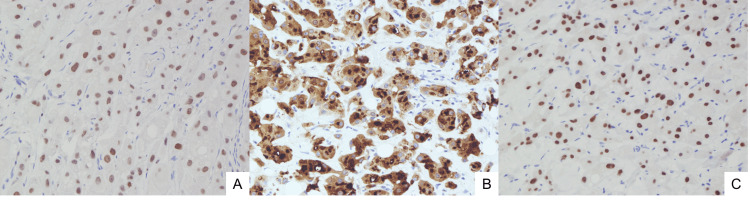
Immunohistochemical findings The tumor cells were positive for A) GATA-3, B) GCDFP-15, and C) androgen receptor.

The lipoma and axillary tumor were excised under local anesthesia for diagnostic evaluation. The axillary lesion was removed in a spindle-shaped manner (Figure 2), and histopathological examination confirmed apocrine carcinoma. The excised specimen demonstrated a nodular lesion measuring 10 × 6 mm, involving the superficial dermis and subcutaneous fat. Microscopically, polygonal atypical cells with eosinophilic granular cytoplasm and large nuclei containing prominent nucleoli were observed. These cells proliferated invasively in trabecular and small nest-like patterns, occasionally forming tubular structures (Figure 3). Lymphatic invasion was also present, and both lateral and deep margins were positive. Immunohistochemical analysis revealed tumor cell positivity for GATA-3, GCDFP-15, and the androgen receptor, while mammaglobin was negative (Figure 4). The Ki-67 labeling index was 5%-10%. Based on these findings, the tumor was diagnosed as apocrine carcinoma originating from cutaneous adnexal glands or accessory breast tissue.

**Figure 5:**
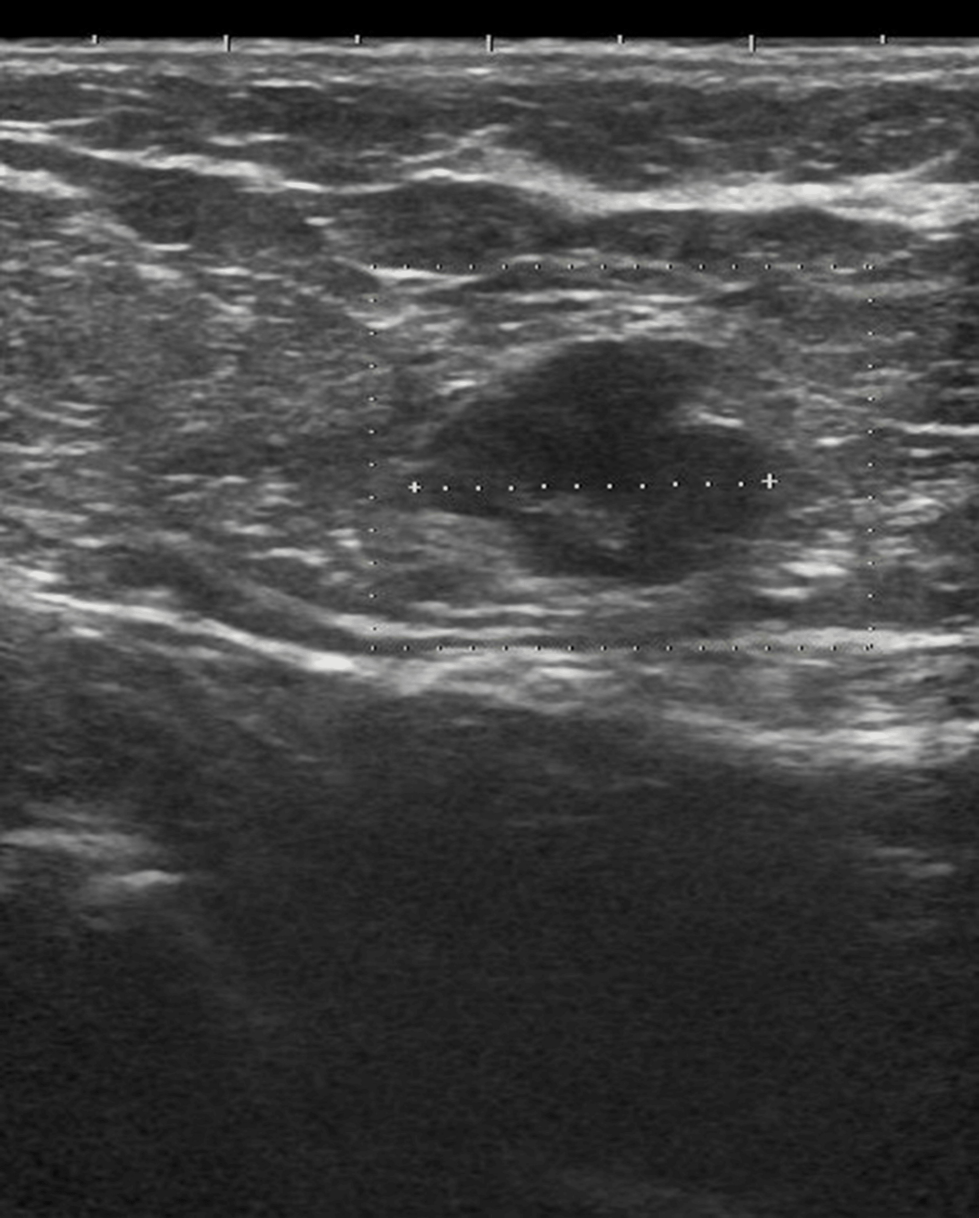
Ultrasonography Ultrasonography indicated a 14-mm lymph node enlargement suspicious for metastasis.

Ultrasonography and contrast-enhanced computed tomography (CT) scans were performed to evaluate metastasis. Ultrasonography demonstrated a 14-mm enlarged lymph node in the axilla, suggestive of metastatic involvement (Figure 5). CT scans revealed no metastatic lesions in the head, chest, or abdomen. Furthermore, no breast lesions were detected on ultrasonography or the CT scans.

**Figure 6:**
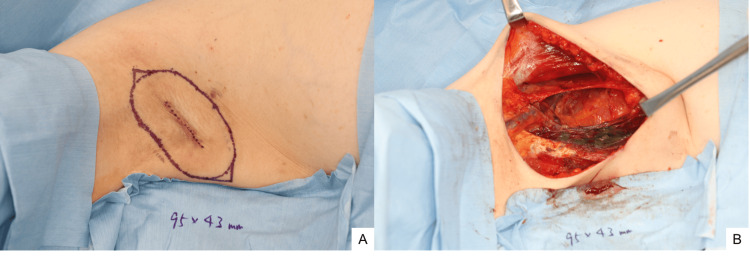
Second surgery A) An additional wide excision was conducted with a 2-cm margin around the previous surgical scar under general anesthesia. B) Axillary lymph node dissection and additional excision beneath the latissimus dorsi fascia were conducted.

Under general anesthesia, a wide excision was conducted with a 2-cm margin around the previous surgical scar, extending to the superficial fascia (Figure 6A). Four enlarged lymph nodes adjacent to the tumor were also removed. Intraoperative frozen section analysis revealed a positive deep margin and metastatic involvement in one of the four lymph nodes. However, subsequent immunohistochemical analysis revealed that the cells present at the deep margin were histiocytes negative for AE1/3, GATA3, and GCDFP-15, but positive for CD68. Accordingly, the deep margin was ultimately determined to be negative. Based on the frozen section findings, axillary lymph node dissection and additional excision beneath the latissimus dorsi fascia were performed, including partial resection of the underlying latissimus dorsi muscle (Figure 6B). The wound was closed with the placement of a suction drain.

**Figure 7:**
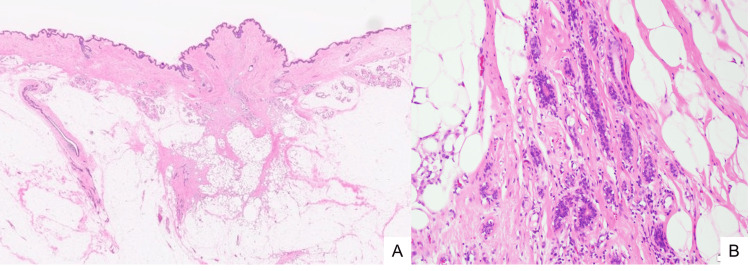
Accessory breast tissue Multiple duct-like glandular structures, consistent with accessory breast tissue, were observed in the subcutaneous adipose tissue adjacent to the surgical scar. A) H&E, low-power view, B) H&E ×200

Histopathological examination revealed multiple duct-like glandular structures consistent with accessory breast tissue within the subcutaneous adipose tissue adjacent to the surgical scar (Figure 7). Based on these findings, a diagnosis of ABAC was confirmed. An 8-mm metastasis was identified in one lymph node, while no metastases were present in the dissected nodes. Additional immunohistochemical staining demonstrated that the carcinoma cells were negative for estrogen and progesterone receptors, with equivocal HER2 expression. Fluorescence in situ hybridization for HER2 was negative, indicating a triple-negative phenotype. The tumor was staged as T1cN1M0 (stage IIA) according to the 8th edition of the AJCC TNM staging system for breast cancer. As no specific staging system is available for accessory breast carcinoma, the breast cancer staging criteria were applied in this case.

**Figure 8:**
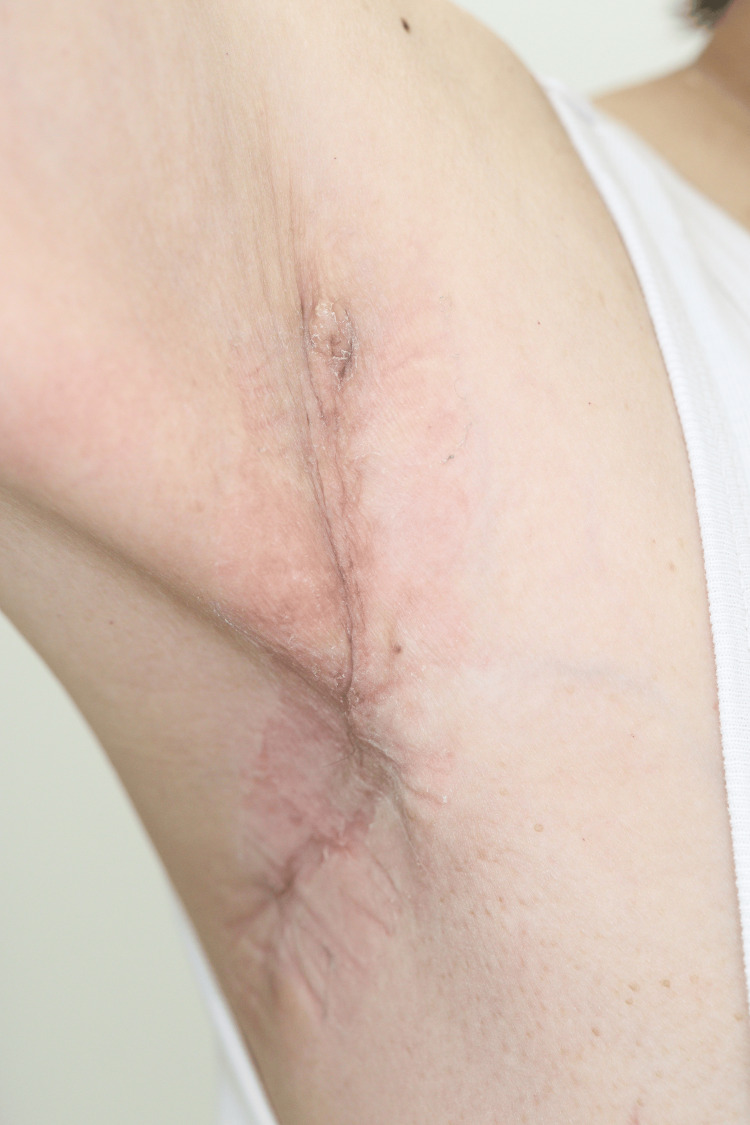
Five-month follow-up No recurrence or lymphedema was observed. Skin irritation due to adhesive tape was observed.

The drain was removed on postoperative day 14; however, a seroma infection developed on postoperative day 18. The wound was partially opened, and the infection was treated using oral antibiotics, wound irrigation, and compression therapy. Postoperative radiotherapy was administered at a total dose of 50 Gy in 25 fractions over six weeks. At the five-month follow-up, no evidence of recurrence or lymphedema was observed (Figure 8). The patient remains under outpatient follow-up.

## Discussion

ABAC is extremely rare. Accessory breast tissue occurs in 0.4 to 6% of women, most frequently in the axilla [3], with the highest incidence reported in the Japanese population [3,9,10]. Carcinoma arising in accessory breast tissue accounts for 0.3 to 0.6% of all breast cancers [4]. Accessory breast carcinoma typically presents as a subcutaneous mass in the axilla [8]. Apocrine carcinoma itself represents about 1% of breast cancer cases [5-7]. Few cases of ABAC have been reported in Japanese-language literature [8], and this is the first reported case of ABAC in the English-language literature.

Differential diagnoses for ABAC include primary cutaneous apocrine carcinoma (PCAC) and cutaneous metastasis from breast cancer [1,2]. Both ABAC and PCAC most commonly develop in the axilla [1]. Histopathologically, ABAC and PCAC are similar, and differentiation requires careful evaluation of the tumor's layer of origin and adjacent tissues [1]. In this case, accessory breast tissue adjacent to the carcinoma supported the diagnosis of ABAC, although PCAC cannot be completely excluded due to the absence of standardized diagnostic criteria. The small mass initially noted by the patient was considered to represent accessory breast tissue. Larger studies are needed to develop standardized diagnostic criteria for ABAC.

There is no standardized treatment for accessory breast carcinoma. Axillary lymph node metastasis occurs in approximately 50% of patients, and wide local excision with lymph node dissection is commonly performed [4,8]. Similarly, PCAC frequently metastasizes to the axillary lymph nodes, and wide local excision with lymph node dissection is also recommended [1]. Sentinel lymph node biopsy (SLNB) has been reported in some cases but is not yet established as a standard procedure [11-13]. In this case, SLNB was not performed due to altered lymphatic drainage from prior excision. Instead, adjacent enlarged lymph nodes were excised, and axillary lymph node dissection was carried out following confirmation of metastasis on intraoperative frozen section analysis. Given the risk of lymphedema associated with lymph node dissection, further studies are needed to evaluate SLNB in the management of accessory breast carcinomas.

Postoperative therapy for accessory breast carcinoma generally follows treatment strategies established for breast cancer [4,8]. Postoperative treatment options include endocrine therapy, chemotherapy, and radiotherapy. Hormone receptor status is assessed histopathologically, and endocrine therapy, such as tamoxifen or aromatase inhibitors, is considered for hormone receptor-positive tumors [8]. More than half of reported cases of accessory breast carcinoma are positive for hormone receptors [8,14]. In contrast, apocrine carcinoma of the breast rarely expresses hormone receptors, with over half of cases demonstrating a triple-negative phenotype [5-7]. The present case was also triple-negative; therefore, endocrine therapy was not administered. Radiotherapy was delivered without chemotherapy, as metastatic disease was limited to a single lymph node. When treating axillary apocrine carcinoma, it is essential to distinguish ABAC from PCAC, since postoperative endocrine therapy may be considered in cases of ABAC.

## Conclusions

We report an extremely rare case of ABAC, an uncommon variant of accessory breast carcinoma. To the best of our knowledge, this is the first case documented in the English-language literature. Although no standardized treatment protocol exists for accessory breast carcinoma, wide local excision with lymph node dissection is generally recommended. Primary apocrine carcinoma of the axilla may arise either from cutaneous adnexal glands or from accessory breast tissue; therefore, careful assessment of the tumor’s site of origin and surrounding structures is essential. Since endocrine therapy may be an option for accessory breast carcinoma, distinguishing accessory breast apocrine carcinoma (ABAC) from primary cutaneous apocrine carcinoma is critical for appropriate management. Although this report describes a single case with limited follow-up, it provides an important clinical message: accessory breast origin should be considered when evaluating apocrine carcinoma in the axilla, as accurate diagnosis directly influences treatment strategy.
